# Susceptibility to chronic immobilization stress‐induced depressive-like behaviour in middle‐aged female mice and accompanying changes in dopamine D1 and GABA_A_ receptors in related brain regions

**DOI:** 10.1186/s12993-021-00175-z

**Published:** 2021-04-16

**Authors:** Guofen Cao, Gaili Meng, Li Zhu, Jie Zhu, Nan Dong, Xiaolan Zhou, Sumei Zhang, Yongai Zhang

**Affiliations:** 1 Xi’an Medical University School of Nursing, Xi’an, Shaanxi PR China; 2 Northwest Women and Children Hospital, Xi’an, Shaanxi PR China; 3 College of Forensic Medicine, Xi’an Jiaotong University Health Science Center, Xi’an, Shaanxi PR China

**Keywords:** Middle‐aged, Depressive‐like behaviour, Chronic immobilization stress, Dopamine, GABA_A_

## Abstract

**Background:**

Middle-aged females, especially perimenopausal females, are vulnerable to depression, but the potential mechanism remains unclear. Dopaminergic and GABAergic system dysfunction is involved in the pathophysiology of depression. In the current study, we used 2-month-old and 11-month-old C57BL/6 mice as young and middle-aged mice, respectively. Chronic immobilization stress (CIS) was used to induce depressive-like behaviour, and the sucrose preference test (SPT), tail suspension test (TST) and forced swim test (FST) were used to assess these behaviours. We then measured the mRNA levels of dopamine receptor D1 (*DRD1*) and the GABA_A_ receptors *GABRA1*, *GABRB2* and *GABRG2* in the nucleus accumbens (NAc) and prefrontal cortex (PFC).

**Results:**

We found that immobility time in the FST was significantly increased in the middle-aged mice compared with the middle-aged control mice and the young mice. In addition, the preference for sucrose water was reduced in the middle-aged mice compared with the middle-aged control mice. However, CIS did not induce obvious changes in the performance of the young mice in our behavioural tests. Moreover, the middle-aged mice exhibited equal immobility times as the young mice in the absence of stress. Decreases in the mRNA levels of *DRD1*, *GABRA1*, and *GABRB2* but not *GABRG2* were found in the NAc and PFC in the middle-aged mice in the absence of stress. Further decreases in the mRNA levels of *DRD1* in the NAc and *GABRG2* in the NAc and PFC were found in the middle-aged mice subjected to CIS.

**Conclusions:**

Our results suggested that ageing could not directly induce depression in the absence of stress. However, ageing could induce susceptibility to depression in middle-aged mice in the presence of stress. CIS-induced decreases in *DRD1* and *GABRG2* levels might be involved in the increase in susceptibility to depression in this context.

## Introduction

Depression has become the leading cause of disability worldwide [1–3]. The incidence of depression is obviously sex-dependent [4, 5]. Females are more than twice as likely to develop depression as males [6]. Moreover, the incidence of depression is obviously age-dependent [5, 7, 8]. During middle age, especially during perimenopause, which occurs in the final years of the female reproductive stage, the rates of major depressive disorder and clinical depression symptoms increase two- to threefold in women [6]. These human data indicate the importance of ageing in susceptibility to depression in middle age in women. However, few studies have investigated the effect of stress exposure and the mechanisms underlying susceptibility to depression in middle age.

The onset of depression is related to changes in various neurotransmitter and receptor levels in the brain [9, 10]. Dopamine (DA) receptor D1 (*DRD1*) has been found to be a functionally specific marker of the DA system [11, 12]. Lower *DRD1* levels are found in patients with affective disorders, including depression [13, 14]. In addition, γ-aminobutyric acid (GABA) is the most abundant inhibitory neurotransmitter and modulates the local neuronal circuitry through acting on its receptors, such as the GABA_A_ receptor [15, 16]. Studies have found abnormally decreased GABA_A_ receptor function in mood disorder patients [17, 18]. In the mammalian brain, *GABRA1*, *GABRB2* and *GABRG2* encode the major GABA_A_ receptor subunits α1, β2 and γ2, respectively [19]. Antidepressants such as imipramine have been reported to increase *GABRA1*, *GABRB2* and *GABRG2* expression at the mRNA level [20]. However, how *DRD1* and the levels of the GABA_A_ receptors *GABRA1*, *GABRB2* and *GABRG2* change in depression in middle age remains unclear.

Chronic immobilization stress (CIS) is a paradigm that is commonly used to study depressive-like behaviours in rodents [21, 22]. Studies have shown that 10- to 14-month-old middle-aged rodents, due to an irregular oestrous cycle or for other reasons, exhibit abnormal emotional behaviours [23–25]. In the current study, we used 2-month-old and 11-month-old C57BL/6 mice as young and middle-aged mice, respectively. After 21 days of CIS exposure, the sucrose preference test (SPT), tail suspension test (TST) and forced swim test (FST) were used to assess changes in stress-induced depressive-like behaviour in middle-aged mice. We then measured the mRNA levels of *DRD1* and the GABA_A_ receptors *GABRA1*, *GABRB2* and *GABRG2* in the nucleus accumbens (NAc) and prefrontal cortex (PFC) to explore the underlying mechanism. Our study identifies a potential neurotransmitter system-related mechanism underlying vulnerability to depression in middle age.

## Materials and methods

### Animals

Female C57BL/6 mice were purchased from Beijing Vital River Laboratory Animal Technology Co., Ltd. In our experiment, 11-month-old mice (weighing approximately 25–32 g) were used as middle-aged mice, and 2-month-old mice (weighing approximately 20–25 g) were used as young control mice. The animals were housed three to four animals per cage in a regulated environment (23 ± 1 °C, 50 ± 5 % humidity) on a 12:12 h light/dark cycle (lights on at 07:00) with food and water available ad libitum. The animals were allowed to habituate to the room for one week before experimental manipulations were performed. The present study was approved by the Ethical Committee of Xi’an Jiaotong University Institutional Animal Care and Use.All procedures were performed according to the National Institutes of Health Guidelines for the Care and Use of Laboratory Animals.

### Experimental procedure

The mice of the two age groups were subjected to an immobilization stress protocol (CIS groups: young CIS, n = 8; middle-aged CIS, n = 10) or a home-cage control protocol (control groups: young con, n = 8; middle-aged con, n = 10) for 21 days (day 1–day 21). After the CIS protocol, all mice underwent behavioural tests, including the SPT (day 21–day 22), FST (day 23) and TST (day 23).

### Chronic immobilization stress (CIS)

To generate a reproducible animal model of stress-induced depressive-like behaviours, we used the CIS protocol based on previous studies [22]. Briefly, the mice in the CIS groups were subjected to immobilization stress by restraint using a plastic cylinder with a diameter of 3 cm and height of 12 cm. The animals were subjected to 6 h of immobilization stress (9:00–15:00) once daily for 21 consecutive days (day 1–day 21).

### Forced swim test (FST)

The FST was conducted between 9:00 and 12:00. The mice were placed in 15 cm of water (24 ± 1℃) in Plexiglas cylinders (25 cm height × 15 cm diameter) for 6 min. The behaviour of the mice was recorded with a video tracking system (SMART 3.0), and immobility was assessed during the last 4 min of the session. The water was replaced after each trial.

### Tail suspension test (TST)

The TST was conducted between 13:00 and 16:00. Each mouse was individually suspended 30 cm above the floor from a fixed hook using a small piece of adhesive tape placed approximately 2 cm from the tip of the tail for 6 min. The behaviour of the mice was recorded with a video tracking system (SMART 3.0), and immobility was assessed during the last 4 min of the session.

### Sucrose preference test (SPT)

To quantitatively evaluate anhedonia, we subjected mice to the SPT by simultaneously presenting a bottle of water and a bottle of 1 % (wt/vol) sucrose solution. The bottles were weighed prior to being placed on the lid of each mouse’s home cage and reweighed to calculate the amount of sucrose solution and water that had been consumed after 24 h. The positions of the bottles were changed every 12 h to ensure that the mice did not develop a preference for one side. Sucrose preference was calculated as the percentage of sucrose solution consumed relative to the total fluid intake: sucrose intake/(sucrose intake + water intake)×100. Four SPT trials were conducted before the mice were subjected to the CIS protocol. The average sucrose preference in the last two of these trials was used as the pre-test data. The SPT test was conducted from 20:00 on day 21 for 24 h.

### Sample preparation

The mice were sacrificed by cervical dislocation on day 24. The whole brains were removed rapidly. The NAc and PFC were dissected out on an ice-cold plate and immediately frozen in liquid nitrogen until use.

### PCR protocol

Total RNA was extracted using an RNAfast1000 Universal RNA Extraction Kit following the instructions of the manufacturer (Pioneer Biotechnology, China). The samples were eluted in RNase-free H_2_O and quantified using an ELx800 Microplate Reader (BioTek, USA). cDNA was synthesized using the PrimeScript 1st Strand cDNA Synthesis Kit (Perfect Real Time) (TaKaRa Biotechnology, Japan). A total of 500 ng of total RNA from each sample was utilized for each reaction, which was performed according to the manufacturer’s suggested parameters (37 °C for 15 min and 85 °C for 5 s). qPCR was performed on the Bio-Rad iQ5 system (Bio-Rad, USA) using SYBR Premix Ex Taq II (TaKaRa Biotechnology, Japan) under the following conditions: 95 °C for 30 s and 95 °C for 10 s, 57 °C for 30 s and 72 °C for 30 s for 40 cycles. *Gapdh* was used as an endogenous control for qPCR, and the relative expression levels were determined by the 2 ^− △△Ct^ method. The primer sequences are shown in Table 1.

**Table 1:** Primer sequences

Primer	Forward (5’-3’)	Reverse (5’-3’)
*DRD1*	TGTGACACGAGGTTGAGC	GGTGGTCTGGCAGTTCTT
*GABRA1*	AGTTTCGGACCAGTTTCAGACCAC	CATAAGGTTGTTTAGCCGGAGCAC
*GABRB2*	GATGGACCTAAGGCGGTATCCAC	GGAAGCTCAATCTTTGTCACTCCTG
*GABRG2*	GCCAGTTGCAATTACACAACTTCC	ACTTCAACAGAACTGCGCTTCC
*GAPDH*	TGTGTCCGTCGTGGATCTGA	TTGCTGTTGAAGTCGCAGGAG

The primer pairs were chosen so that the melting temperature (Tm) was between 50 and 65 °C

### Data analysis

All data were presented as the mean ± SEM. Statistical analysis was performed using SPSS 16.0. Statistical differences among most data were analysed by two-way analysis of variance (ANOVA) followed by a post hoc multiple comparisons test (least significant difference, LSD). Differences between pre-test and test data from the SPT were analysed by independent-samples *t* test. *P* < 0.05 was considered statistically significant.

## Results

### CIS-induced susceptibility to depressive-like behaviour in middle-aged mice

**Fig. 1:**
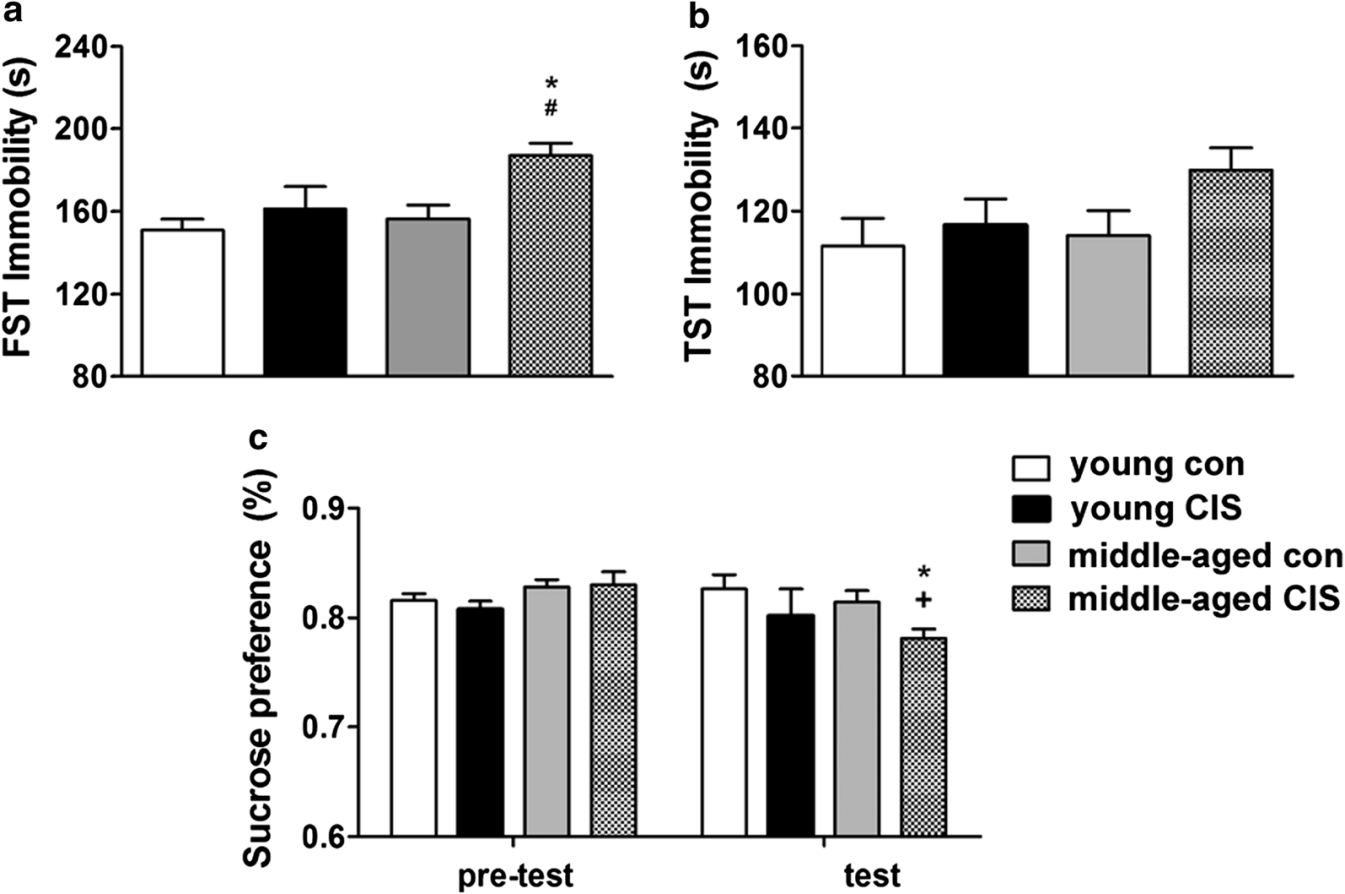
Chronic immobilization stress (CIS)-induced susceptibility to depressive-like behaviour in middle-aged mice. The data are presented as the mean ± SEM. **a** Forced swim test (FST). **b** Tail suspension test (TST). **c** Sucrose preference test (SPT). Age could not directly induce depressive-like behaviour in mice in the absence of stress. However, age could increase the susceptibility to depressive-like behaviour in middle-aged mice upon exposure to CIS (n = 8–10 per group). *P < 0.05 compared with the age-matched control group; #*P* < 0.05 compared with young mice subjected to the same treatment; + *P* < 0.05 compared with the pre-test data of the same group. Differences among the four groups were analysed by two-way analysis of variance (ANOVA) followed by the LSD post hoc multiple comparisons test. Differences between pre-test and test data in the SPT were analysed by independent-samples *t* test

There was a significant effect of age [*F*(1, 32) = 4.582, *P* < 0.05] and CIS exposure [*F*(1, 32) = 7.810, *P* < 0.05] but not an interaction effect [*F*(1, 32) = 1.896, *P* > 0.05] on performance in the FST. Multiple comparisons tests were further conducted, and the results were as follows. No difference in immobility time was found between the two control groups of mice. CIS had no effect on immobility in the two young groups of mice, as indicated by the similar immobility times in the FST. However, regarding the two middle-aged groups of mice, the immobility time was significantly longer in the middle-aged CIS group than the middle-aged control group (Fig. 1a, P < 0.05). Furthermore, regarding the two CIS-treated groups of mice, the middle-aged CIS group exhibited a longer immobility time than the young CIS group (Fig. 1a, P < 0.05). The same tendency was found for the TST. There was no effect of age [*F*(1, 32) = 1.661, *P* > 0.05], CIS exposure [*F*(1, 32) = 2.892, *P* > 0.05] or their interaction [*F*(1, 32) = 0.765, *P* > 0.05] on performance in the TST. The immobility time was longer in the middle-aged CIS group than in the middle-aged control group and the young CIS group, although the difference failed to reach statistical significance (Fig. 1b, CIS effect, *P* = 0.099). All mice performed equally in the SPT before CIS exposure (Fig. 1c). The results showed a significant effect of CIS exposure [*F*(1, 32) = 4.226, *P* < 0.05] but not age [*F*(1, 32) = 1.325, *P* > 0.05] or the interaction between these factors [*F*(1, 32) = 0.081, *P* > 0.05]. Multiple comparisons tests revealed that the preference for sucrose water was significantly reduced in the middle-aged CIS group compared to the middle-aged control group (Fig. 1c, P < 0.05). Moreover, an independent-samples *t* test revealed that the preference for sucrose water was significantly reduced compared to the pre-test level in the middle-aged CIS group (Fig. 1c, t = − 3.291, df = 18, *P* < 0.05). These results indicated that ageing could not directly induce depressive-like behaviour in mice in the absence of stress. However, ageing increased the susceptibility to depressive-like behaviour in mice upon exposure to CIS.

### Changes in the mRNA level of DRD1 in the NAc and PFC

**Fig. 2:**
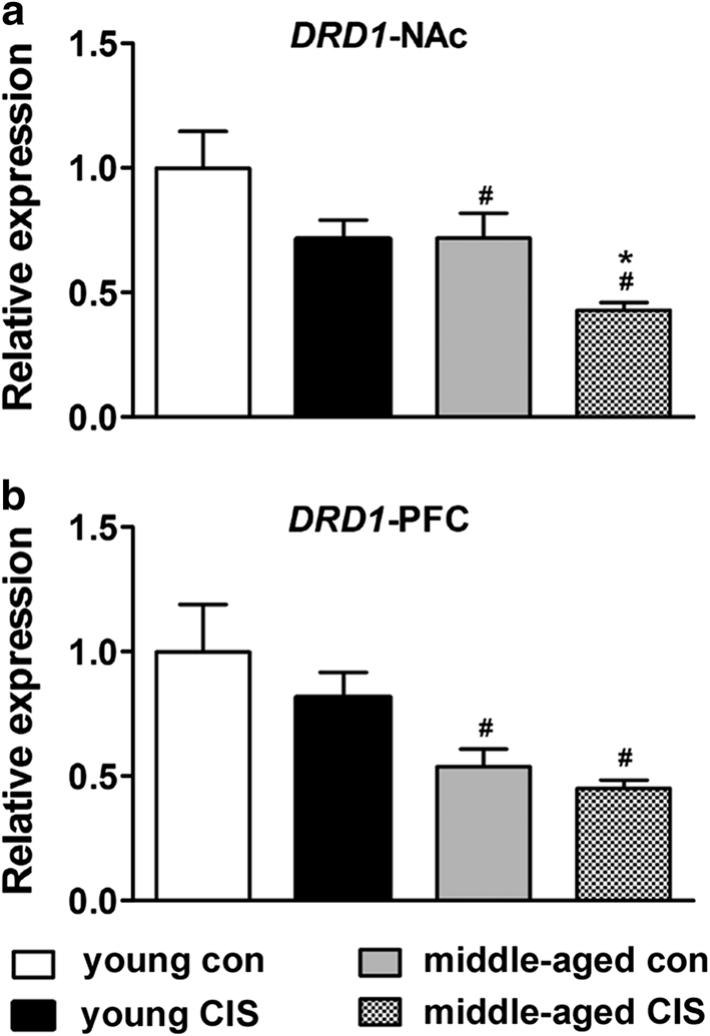
Changes in the mRNA level of *DRD1* in the nucleus accumbens (NAc) and prefrontal cortex (PFC). The data are presented as the mean ± SEM. **a** *DRD1* expression in the NAc. **b** *DRD1* expression in the PFC. Age could directly induce a decrease in the mRNA level of *DRD1* in the NAc and PFC in mice in the absence of stress. A further decrease in the mRNA level of *DRD1* in the NAc was found in middle-aged mice upon exposure to CIS. *P < 0.05 compared with the age-matched control group; # *P* < 0.05 compared with young mice subjected to the same treatment. Two-way ANOVA followed by the LSD post hoc multiple comparisons test

Analysis of the change in the mRNA level of DRD1 in the NAc revealed an effect of age [F(1, 32) = 9.167, P < 0.05] and CIS exposure [F(1, 32) = 9.392, P < 0.05] but not their interaction [F(1, 32) = 0.001, P > 0.05]. Multiple comparisons tests were further conducted, and the results were as follows. There was a significant decrease in the mRNA level of *DRD1* in both the middle-aged control group and middle-aged CIS group (Fig. 2a, P < 0.05) compared with the young control and young CIS groups. Furthermore, there was a significant decrease in the mRNA level of *DRD1* in the middle-aged CIS group (Fig. 2a, P < 0.05) compared with the middle-aged control group. However, CIS had no effect on the mRNA level of *DRD1* in the two young groups. Analysis of the change in the mRNA level of *DRD1* in the PFC revealed a significant effect of age [F(1, 32) = 15.741, P < 0.05] but not CIS exposure or the interaction between these two factors. There was a significant decrease in the mRNA level of *DRD1* in both the middle-aged control group and middle-aged CIS group (Fig. 2b, P < 0.05) compared with the young control and young CIS groups. However, CIS had no effect on the mRNA level of *DRD1* in the young mice or the middle-aged mice. Taken together, these results suggest that ageing could directly induce a decrease in the mRNA level of *DRD1* in the NAc and PFC in mice in the absence of stress. In addition, a further decrease in the mRNA level of *DRD1* in the NAc was found in middle-aged mice upon exposure to CIS, which might have been involved in the increase in the susceptibility of middle-aged mice to depressive-like behaviour.

### Changes in the mRNA level of GABRG2 in the NAc and PFC

**Fig. 3:**
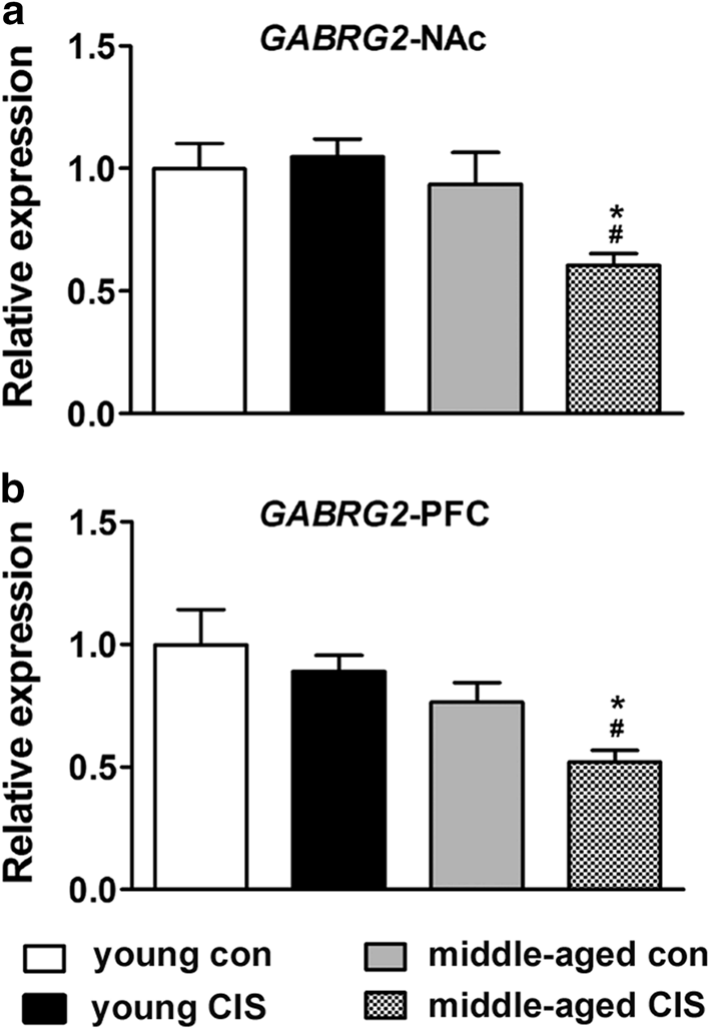
Changes in the mRNA level of *GABRG2* in the nucleus accumbens (NAc) and prefrontal cortex (PFC). The data are presented as the mean ± SEM. **a** *GABRG2* expression in the NAc. **b** *GABRG2* expression in the PFC. Age had no obvious effect on the mRNA level of *GABRG2* in the NAc and PFC in mice in the absence of stress. However, a decrease in the mRNA level of *GABRG2* in the NAc and PFC was found in middle-aged mice upon exposure to CIS. *P < 0.05 compared with the age-matched control group; # *P* < 0.05 compared with young mice subjected to the same treatment. Two-way ANOVA followed by the LSD post hoc multiple comparisons test

Analysis of the change in the mRNA level of *GABRG2* in the NAc revealed a significant effect of age [F(1, 32) = 7.07, P < 0.05] but no other effects. Multiple comparisons tests were further conducted, and the results were as follows. No difference was found between the two control groups. In addition, no difference was found between the two young groups. However, the mRNA level of *GABRG2* in the middle-aged CIS group was significantly decreased compared with that in the young CIS group (Fig. 3a, P < 0.05). Moreover, the mRNA level of *GABRG2* in the middle-aged CIS group was decreased significantly compared with that in the middle-aged control group (Fig. 3a, P < 0.05). Similar to analysis of the change in the *GABRG2* level in the NAc, analysis of the change in the mRNA level of *GABRG2* in the PFC revealed a significant effect of age [F(1, 32) = 12.244, P < 0.05] and CIS exposure (F(1, 32) = 4.201, P < 0.05) but not their interaction. No difference was found in *GABRG2* mRNA levels in the PFC between the two control groups or the two young groups. However, the mRNA level of *GABRG2* in the middle-aged CIS group was decreased significantly compared with that in the young CIS group (Fig. 3b, P < 0.05) and the middle-aged control group (Fig. 3b, P < 0.05). Taken together, these results suggest that ageing had no obvious effect on the mRNA level of *GABRG2* in the NAc and PFC in mice in the absence of stress. However, a decrease in the mRNA level of *GABRG2* in the NAc and PFC was found in middle-aged mice upon exposure to CIS, which might have been involved in the increase in the susceptibility of middle-aged mice to depressive-like behaviour in our experiment.

### Changes in the mRNA level of GABRA1 in the NAc and PFC

**Fig. 4:**
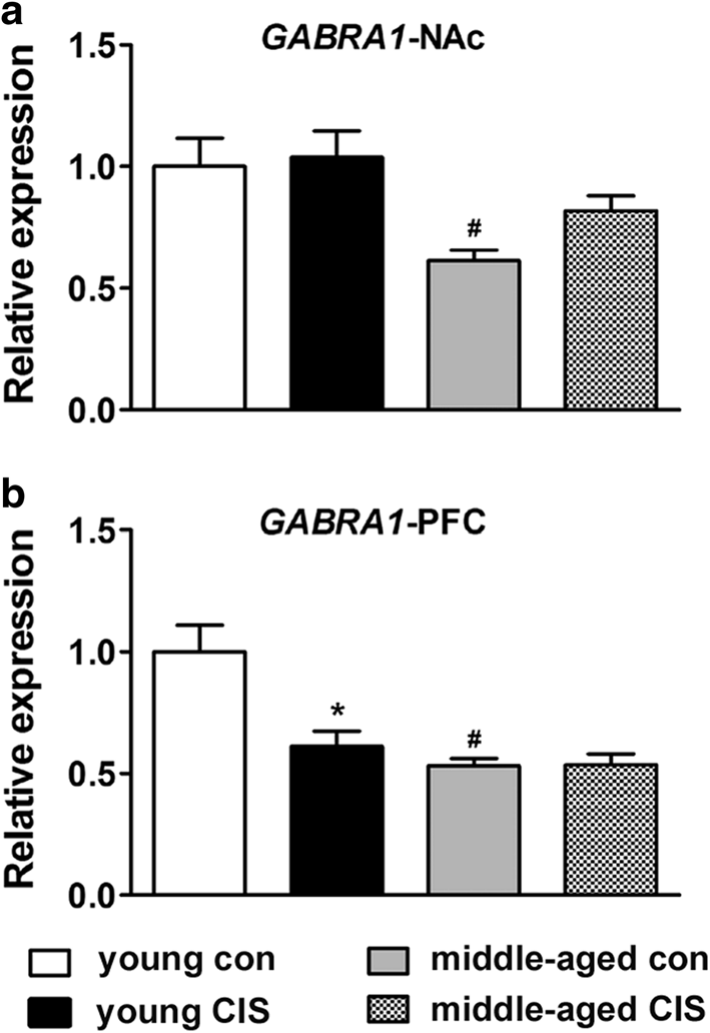
Changes in the mRNA level of *GABRA1* in the nucleus accumbens (NAc) and prefrontal cortex (PFC). The data are presented as the mean ± SEM. **a** *GABRA1* expression in the NAc. **b** *GABRA1* expression in the PFC. Age could directly induce a decrease in the mRNA level of *GABRA1* in the NAc and PFC in mice in the absence of stress. Moreover, CIS could induce a reduction in the mRNA level of *GABRA1* in the PFC in young mice but not middle-aged mice. *P < 0.05 compared with the age-matched control group; #*P* < 0.05 compared with young mice subjected to the same treatment. Two-way ANOVA followed by the LSD post hoc multiple comparisons test

Analysis of the change in the mRNA level of *GABRA1* in the NAc revealed a significant effect of age [F(1, 32) = 13.600, P < 0.05] but no other effects. There was a significant decrease in the mRNA level of *GABRA1* in the middle-aged control group compared with the young control group (Fig. 4a, P < 0.05). There was also a decrease in the mRNA level of *GABRA1* in the middle-aged CIS group, although the difference failed to reach statistical significance (Fig. 4a, P = 0.068, compared with young CIS group). However, CIS had no obvious effect on the mRNA level of *GABRA1* in young mice or middle-aged mice. In the PFC, analysis of the change in the mRNA level of *GABRA1* revealed significant effects of age [F(1, 32) = 18.563, P < 0.05], CIS exposure [F(1, 32) = 9.227, P < 0.05] and their interaction [F(1, 32) = 9.509, P < 0.05]. As shown in Fig. 4b, there was a significant decrease in the mRNA level of *GABRA1* in the middle-aged control group compared with the young control group (Fig. 4b, P < 0.05). In addition, there was a significant decrease in the mRNA level of *GABRA1* in the young CIS group compared with the young control group (Fig. 4b, P < 0.05). However, CIS had no obvious effect on the mRNA level of *GABRA1* in middle-aged mice. These results indicated that ageing could directly induce a decrease in the mRNA level of *GABRA1* in the NAc and PFC in mice in the absence of stress. Moreover, a reduction in the mRNA level of *GABRA1* in the PFC might be involved in stress-induced neurobiological changes in young individuals, although no difference in immobility time was observed in our experiment.

### Changes in the mRNA level of GABRB2 in the NAc and PFC

**Fig. 5:**
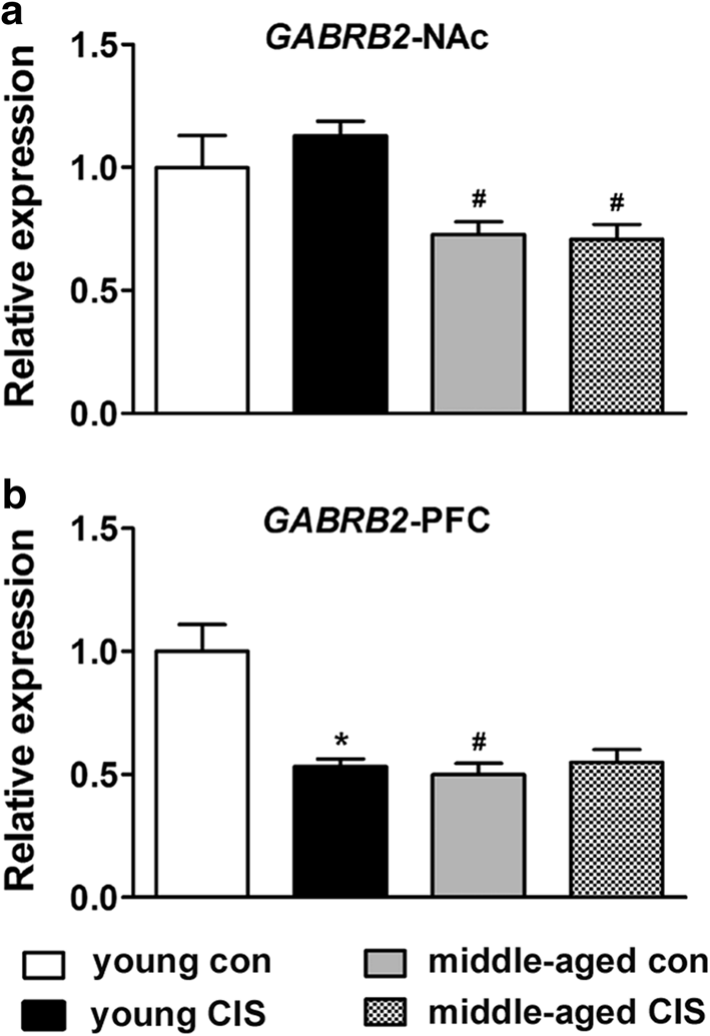
Changes in the mRNA level of *GABRB2* in the nucleus accumbens (NAc) and prefrontal cortex (PFC). The data are presented as the mean ± SEM. **a** *GABRB2* expression in the NAc. **b** *GABRB2* expression in the PFC. Age could directly induce a decrease in the mRNA level of *GABRB2* in the NAc and PFC in mice in the absence of stress. Moreover, CIS could induce a reduction in the mRNA level of *GABRB2* in the PFC in young mice but not middle-aged mice. * *P* < 0.05 compared with the age-matched control group; # *P* < 0.05 compared with young mice subjected to the same treatment. Two-way ANOVA followed by the LSD post hoc multiple comparisons test

Analysis of the change in the mRNA level of *GABRB2* in the NAc revealed a significant effect of age [F(1, 32) = 19.932, P < 0.05] but no other effects. The mRNA expression of *GABRB2* showed a similar pattern as that of *GABRA1*. As shown in Fig. 5a, there was a significant decrease in the mRNA level of *GABRB2* in the middle-aged control group compared with the young control group (Fig. 5a, P < 0.05). There was also a significant decrease in the mRNA level of *GABRB2* in the middle-aged CIS group (Fig. 5a, P < 0.05, compared with young CIS group). However, CIS had no obvious effect on the mRNA level of *GABRB2* in the young mice or the two middle-aged mice. In the PFC, *GABRB2* also exhibited a similar expression pattern as *GABRA1*. Analysis of the change in the mRNA level of *GABRB2* revealed a significant effect of age [F(1, 32) = 14.324, P < 0.05], CIS exposure [F(1, 32) = 10.751, P < 0.05] and their interaction [F(1, 32) = 16.493, P < 0.05]. As shown in 5b, there was a significant decrease in the mRNA level of *GABRB2* in the middle-aged control group compared with the young control group (Fig. 5b, P < 0.05). In addition, there was a significant decrease in the mRNA level of *GABRB2* in the young CIS group compared with the young control group (Fig. 5b, P < 0.05). However, CIS had no obvious effect on the mRNA level of *GABRB2* in the two middle-aged mice. These results indicated that ageing could directly induce a decrease in the mRNA level of *GABRB2* in the NAc and PFC in mice in the absence of stress. Moreover, a reduction in the mRNA level of *GABRB2* in the PFC might be involved in stress-induced neurobiological changes in young individuals, although no difference in immobility time was observed in our experiment.

## Discussion

Ageing causes a high incidence of depression. In middle age, females are susceptible to depression [6, 26]. In the current study, we used 2-month-old and 11-month-old mice as the young and middle-aged mice, respectively, to study changes in stress-induced depressive-like behavioural in middle-aged mice. We found a significant increase in immobility time in the middle-aged CIS group compared to the middle-aged control group (Fig. 1a) and the young CIS group (Fig. 1a). In addition, there was a significant reduction in preference for sucrose water in the middle-aged CIS group compared to the middle-aged control group (Fig. 1c). However, CIS did not induce obvious changes in the performance of the young mice in our behavioural tests (Fig. 1). Furthermore, we found no differences in performance on our behavioural tests between the two control groups in our experiment (Fig. 1). These results indicated that ageing per se could not directly induce depressive-like behaviour in mice in the absence of stress. However, ageing could induce susceptibility to depressive-like behaviour in middle-aged mice upon exposure to stress. The precise mechanisms are poorly understood.

Notably, studies have shown that from 10 to 14 months of age, irregular oestrous cycles are found in spontaneously ageing female rodents; this period has been defined as perimenopause. A study reported that, the behaviour of perimenopausal mice not exposed to stress in the FST is the same as that of young adult mice [25]. Our behavioural results were consistent with this finding in that the middle-aged control mice did not display differences compared to the young control group. However, in the presence of stress, depression susceptibility is increased in perimenopausal humans and rodents compared to young individuals [6, 24]. Therefore, perimenopause might have played a potential role in the behavioural changes in middle-aged mice in our study. Increasing evidence suggests that fluctuations in ovarian hormones and neurosteroids derived from these hormones can result in altered GABAergic function [27, 28]. Specifically, the GABA_A_ receptor can be modulated by allopregnanolone (3α-hydroxy-5α-pregnan-20-one; ALLO), a metabolite of progesterone, via dose-dependent enhancement of GABA-induced Cl-ion channels [29, 30]. During perimenopause, a shift in the level of ALLO might induce the failure of the GABA_A_ receptor to regulate dopamine and other neurotransmitters. These changes further result in hypothalamic-pituitary-adrenal (HPA) axis dysfunction, including changes in the extent and duration of HPA axis activation during stress responses, and thereby increase vulnerability to depression in perimenopause [6, 18, 31]. Thus, the GABA_A_ receptor and neurotransmitters that are regulated by GABA_A_ receptors might play an important role in stress-induced depression susceptibility in middle-aged mice.

GABA is the most abundant inhibitory neurotransmitter and is involved in the regulation of various neuronal activities. DA neurons are mainly located in the ventral tegmental area (VTA) [32]. Importantly, it has been estimated that 50-70 % of all afferents to VTA DA neurons are GABAergic, and these inhibitory inputs have a major impact on the activity of DA neurons [33]. DA projections from the VTA to the NAc, which constitute the mesolimbic DA system [32, 34], and DA projections from the VTA to the PFC, which constitute the mesocortex DA system [32], play important roles in motivated behaviours [35] and depression [36]. Evidence has shown that stress can increase DA projections from the VTA to the NAc and PFC [37, 38]. However, after the period of stress, there is an approximately 50 % reduction in dopaminergic neuron activity as well as the levels of DA receptors such as *DRD1* [39, 40]. In addition, Yang et al. reported that NAc subnuclei regulate VTA DA subpopulations via GABA receptors, generating direct inhibitory and disinhibitory effects [41]. In the current study, we found that in the absence of stress, the mRNA levels of *DRD1*, *GABRA1* and *GABRB2* were decreased significantly in middle-aged mice (middle-aged control group) in both the NAc (Figs. 2a, 4a and 5a) and PFC (Figs. 2b, 4b and 5b) (compared with the young control group). CIS induced no further change in the mRNA levels of *GABRA1* and *GABRB2*. However, there was a decrease in the mRNA level of *DRD1* in the middle-aged group compared with the middle-aged control group in the NAc (Fig. 2b). Comparatively, the mRNA level of *GABRG2* showed no obvious change in either the NAc (Fig. 3a) or PFC (Fig. 3b) in middle-aged mice in the absence of stress. However, CIS induced a decrease in the mRNA level of *GABRG2* in both the NAc (Fig. 3a) and PFC (Fig. 3b) in middle-aged mice. Based on the current study, we speculated that ageing could induce a decrease in *GABRA1* and *GABRB2* levels in the NAc and PFC in middle-aged individuals. The combined effects of CIS and ageing induced a decrease in *GABRG2* levels in the NAc and PFC. Decreases in GABA_A_ receptor levels might further reduce the levels of DRD1 in the NAc but not the PFC through projections. Thus, CIS-induced decreases in *DRD1* and *GABRG2* levels might be involved in susceptibility to depression in middle-aged female mice. Additionally, CIS induced significant decreases in the mRNA levels of *GABRA1* (Fig. 4b) and *GABRB2* (Fig. 5b) in the PFC in young mice. However, in our behavioural tests, no differences were found between the two young groups. Preclinical evidence has indicated that the PFC of female mice is more sensitive than that of male mice to the effects of stress [42]. The findings suggested that exposure to stress can lead to sex-specific alterations in prefrontal GABAergic signalling, which contributes to the onset of depressive behaviours in a sex-specific manner [42].

In addition, the following limitations existed in the current study. One limitation is that several other dopamine receptors (such as DRD2 and DRD3), as well as other GABA_A_ receptor subunits (such as α2, α3, β1, and δ), are also obviously involved in the regulation of depression. Further examinations are needed to detect the changes in these molecules. Another limitation is that PCR itself has certain methodological limitations, such as a lack of precision and a lack of raw data. We will use western blotting to study the differences in the expression of these markers in the PFC and the NAc in future studies.

In summary, our results suggested that ageing could not directly induce depression in the absence of stress. However, ageing could induce susceptibility to depression in middle-aged individuals in the presence of stress. CIS-induced decreases in *DRD1* and *GABRG2* levels might be involved in the increase in susceptibility to depression in this context.

## Data Availability

The datasets used and/or analyzed during the current study are available from the corresponding author on reasonable request.

## Abbreviations

DRD1: Dopamine receptor D1
NAc: Nucleus accumbens
PFC: Prefrontal cortex
DA: Dopamine
GABA: γ-Aminobutyric acid
CIS: Chronic immobilization stress
FST: Forced swim test
SPT: Sucrose preference test
TST: Tail suspension test
HPA: Hypothalamic-pituitary-adrenal
ALLO: Allopregnanolone
VTA: Ventral tegmental area
